# The impact of the patient’s initial NACA score on subjective and physiological indicators of workload during pre-hospital emergency care

**DOI:** 10.1371/journal.pone.0202215

**Published:** 2018-08-09

**Authors:** Frederick Schneider, Jan Martin, Gerhard Schneider, Christian M. Schulz

**Affiliations:** Department of Anesthesiology, Klinikum rechts der Isar, School of Medicine, Technical University of Munich, München, Germany; Cleveland Clinic Lerner College of Medicine of Case Western Reserve University, UNITED STATES

## Abstract

**Background:**

Excessive workload may impair patient safety. However, little is known about emergency care providers’ workload during the treatment of life-threatening cases including cardiopulmonary resuscitation (CPR). Therefore, we tested the hypothesis that subjective and physiological indicators of workload are associated with the patient’s initial NACA score and that workload is particularly high during CPR.

**Methods:**

NASA task load index (NASA-tlx) and alarm codes were obtained for 216 sorties of pre-hospital emergency medical care. Furthermore, initial NACA scores of 140 patients were extracted from the physicians’ protocols. The physiological workload indicators mean heart rate (HR) and permutation entropy (PeEn) were calculated for 51 sorties of primary care. General linear mixed models were used to analyze the association of NACA scores with subjective (NASA-tlx) and physiological (mean HR, PeEn) measures of workload.

**Results:**

In contrast to the physiological variables PeEn (p = 0.10) and HR (p = 0.19), the mental (p<0.001) and temporal demands (p<0.001) as well as the effort (p<0.001) and frustration (p = 0.04) subscale of the NASA-tlx were significantly associated with initial NACA scores. Compared to NACA = I, an initial NACA score of VI (representing CPR) increased workload by a mean of 389.5% (p = 0.001) in the mental and 345.9% (p<0.001) in the temporal demands, effort by a mean of 446,8% (p = 0.002) and frustration by 190.0% (p = 0.03). In line with the increase in NASA-tlx, PeEn increased by 20.6% (p = 0.01) and HR by 6.4% (p = 0.57).

**Conclusions:**

Patients’ initial NACA scores are associated with subjective workload. Workload was highest during CPR.

## Introduction

The treatment of emergency patients can lead to occupational stress and job strain.[1] As in emergency medicine, anesthesia is a field in which intense focus and quick decisions are essential. Hence, various instruments to measure workload have been extensively studied in the environment of anesthesia. In a review by Leedal and Smith, different methodological approaches to indicators of anesthetists’ *subjective* and *physiological* workload in the operating theatre were described.[2] Leedal and Smith emphasized the importance of the NASA task load index (NASA-tlx) as an instrument for subjective workload measurements.[2] The NASA-tlx is a multi-dimensional questionnaire that has originally been designed by Hart and Staveland for the National Aeronautics and Space Agency (NASA) to assess pilots’ workload during flight simulations;[3] it has been used in various medical environments[4–6] including anesthesia.[7] Recently, Parsons and co-workers used the NASA-tlx to assess workload during pediatric resuscitation.[8]

Besides subjective workload evaluation, physiological indicators have proven to be valuable correlates of anesthetists’ workload in the operation theatre.[9] In addition to heart rate itself (mean HR),[9–11] heart rate variability (HRV) metrics have been shown to quantify workload in experimental settings and under real-life circumstances.[9, 12–15] Among these, the non-linear HRV metric Permutation Entropy (PeEn) has been found to be a sensible correlate of workload in anesthetists providing general anesthesia.[9] Our recent work indicated that non-linear HRV metrics can also distinguish workload levels during pre-hospital emergency care.[15] However, though excessive workload may impair patient safety,[16–18] data on workload during pre-hospital care including cardiopulmonary resuscitation (CPR) are still scarce.

An additional concern is a potential effect of the alarm code on the physician’s strain prior to the arrival at the scene. Usually, getting valid information about the patient’s situation or the emergency scene previous to arrival is hard to achieve in pre-hospital emergency care.[19] Nevertheless, the intention of briefing alarm-codes is to provide the emergency physician with some information. As this information itself (being valid or not) itself may cause strain, we speculate that the identification of specific alarm codes causing particular strain may help to define training targets.

In order to provide further evidence for the validity of workload measures in pre-hospital emergency care we tested the hypothesis that subjective and physiological indicators of workload are associated with the patient’s initial NACA scores and that workload is particularly high during CPR. Furthermore, we analyzed the physician’s strain related to specific alarm-codes.

## Methods

### Study design

This observational, exploratory study took part during a period of 5 months. 20 physicians completed queries after each sortie in order to evaluate their subjective workload during their shifts as physicians providing pre-hospital emergency care. Furthermore, a chest-belt (Zephyr BioHarnessTM 3, Zephyr Technology Corp., Annapolis, MD, USA) was used to gather electrocardiograms (ECG) during their shifts. All physicians were specialized in emergency medicine; a supplementary qualification that every physician in Germany may obtain after two years of clinical work including at least 6 months in intensive care medicine or 12 months in anesthesia or the emergency department.[20]

The local Ethics Committee at Klinikum rechts der Isar, Technische Universität München approved the study (N° 5771/13; May 11^th^, 2015). Written informed consent was obtained from all participants and all data concerning the participating physicians as well as their patients were raised anonymously. No limitations regarding food intake, activity, sleep or medication were given. During the shift changes no study personnel was present; the emergency physicians filled in the query and put on the chest-belt themselves. Parts of the results, specifically the results regarding the validity of linear and non-linear HRV metrics, have been published earlier.[15]

### Data collection

In Germany, a system of pre-hospital physician-based emergency care is established. In cases where a life-threatening injury or severe sickness (according to a catalogue of certain keywords) is likely, the rescue coordination center sends an emergency physician response vehicle to the scene.[21] This vehicle is staffed with an emergency physician and a paramedic.

From July to November 2015 the emergency physicians were asked to fill in a query after each sortie. The query contained the NASA-tlx,[3] alarm time and date and the alarm code. Pursuant to the physicians’ emergency protocols, the timestamps for alarm, arrival at the patient and handover of the patient at the admitting emergency ward as well as initial NACA scores for each patient were obtained. The sorties took place in a suburban environment in Southern Germany.

After the study period, all participating physicians were asked to complete a survey on their strain related to the alarm codes that they were confronted with during their duties as emergency physicians. A detailed description on the processing of the ECG raw data gathered from the chest-belt and the computation of HRV metrics is provided elsewhere.[15]

### Use of the NACA scoring-system for patient characteristics

The National Advisory Committee for Aeronautics (NACA, a precursor institution of today’s NASA) score is a scoring system that assesses an emergency patient’s severity of injury or illness by eight levels.[22, 23] After it has been originally introduced as a scoring system for trauma patients 24h after admission to a hospital, the modified NACA score presented by Tryba and colleagues in 1980 can be used for pre-hospital injury severity assessment as well. [23, 24] The grades of the NACA score and their verbal descriptions (used in their German translation for this study) are provided in Table 1. As the category NACA 0 (‘No injury or disease’) is not part of the emergency physician’s protocols, it has not been included. Also, cases with an initial NACA score of VII (death of the patient) were excluded. The NACA score well predicts the mortality and the need for ventilation therapy of patients and has substantial inter-rater reliability.[22–24]

**Table 1:** Verbal description of the NACA score’s categories.

Category	Description
**NACA I**	No intervention by an emergency physician necessary
**NACA II**	Slight disturbance, intervention necessary, case can be handled ambulatory
**NACA III**	Moderate to severe, but not life-threatening disturbance, stationary treatment required
**NACA IV**	Development of life-threatening condition cannot be excluded, Intervention by an emergency physician necessary, transport to resuscitation area
**NACA V**	Acute danger for the patient, rapid development to a live-threatening injury possible
**NACA VI**	Life threatening injury, respiratory or cardiac arrest with CPR during the sortie

Verbal descriptions of the NACA sore’s categories. Simplified version adapted from Alessandrini H, Oberladstätter D, Trimmel H, Jahn B, Baubin M. NACA-Scoringsystem. Notfall + Rettungsmedizin 2012; **15**: 42–50. Abbreviations: CPR, cardiopulmonary reanimation.

### Evaluation of subjective workload via NASA task load index

In line with prior work in the field of anesthesia,[9] a six-dimensional version of the NASA-tlx was used for the evaluation of subjective workload; it included the categories *mental demands*, *physical demands*, *temporal demands*, *performance*, *effort*, and *frustration*.[25] Each rating scale ranges from 0 to 20; the steps do not have an additional description. For the study a German transcription of the simplified paper and pencil version of the NASA-tlx provided on the online appearance of the NASA was used.[26] An English translation of the query as well as the German version used for the study are provided in the supplementary material (S1 Text).

### Physiological workload indicators

Based on the times gathered from the physician’s protocols, the timespan from the physician’s arrival at the patient to the handover of the patient at the admitting emergency ward was identified. Due to their high potential to differentiate workload in emergency medicine[15] as well as in anesthetists providing general anesthesia,[9] PeEn and mean HR were included in the analysis[9, 27] of these ECG segments.

### Evaluation of physician pre-sortie anxiety related to the alarm code

The physicians were asked to complete a survey on their strain when reading a certain alarm code. Therefore, a query presented all alarm codes that occurred during the study period (N = 99) in a randomized order. The physicians were asked to rate each alarm code. The Likert scale ranged from ‘not nervous at all’ (1) to ‘very nervous’ (5).

### Statistical analysis

To evaluate the NASA-tlx, descriptive statistics for the NASA-tlx scores and the physician’s physiological workload correlates according to NACA scores were computed. It was accounted for repeated measurements within subjects. Thus, general linear mixed models (GLMM) were used to explore differences among the subjective and physiological workload correlates. In line with our own prior publications,[9, 15] as well as the results of other researchers in the field,[28, 29] the analysis was not adjusted for experience, sex and age. Statistical analysis was performed using the software package SPSS Statistics 24.0.0.0 (IBM Corp. Armonk, NY, USA) and statistical significance was defined as p < 0.05.

## Results

In total, twenty emergency physicians took part in this study (16 male, 4 female). NASA-tlx was obtained during 216 sorties by 17 physicians. Due to missing data in the protocols, NACA scores of only 140 cases were included. The age of the emergency physicians ranged from 30 to 52 years (median 38 years). All physicians were experienced in pre-hospital emergency care; that means they worked as emergency physicians once or twice a month for a median of 6.8 years (minimum 0.9 years; maximum 15.7 years).

### Association of the emergency physician’s subjective and physiological workload indicators with patient’s initial NACA scores

Table 2 provides descriptive statistics for the NASA-tlx, Table 3 for mean HR and PeEn, respectively, according to NACA scores. For the sections *temporal demands* and *effort* three queries had to be excluded due to missing marks in the questionnaire. In the GLMM the *mental* (p<0.001) and *temporal demands* (p<0.001) as well as the *effort* (p<0.001) and *frustration* (p = 0.04) subscale of the NASA-tlx were significantly associated with the patient’s initial NACA score (Table 4). Furthermore, for both subjective and physiological indicators of workload, coefficients were highest for the treatment of patients with NACA VI (CPR).

**Table 2:** Descriptive statistics for the parameters of the NASA task load index.

	Patients initial NACA score					
	I	II	III	IV	V	VI
N	6	29	51	30	11	7
	Median (IQR)	Median (IQR)	Median (IQR)	Median (IQR)	Median (IQR)	Median (IQR)
**Mental**	2 (1–7)	2 (1–5)	3 (1–6)	7.5 (4–14.25)	4 (1–8)	14 (6–19)
**Physical**	1 (1–6.75)	1 (1–3)	1 (1–5)	2 (1–5.25)	3 (1–10)	6 (1–14)
**Temporal**	3 (1–10.25)	1 (1–5)	1 (1–5)	9 (2.75–16)	6 (1–14)	19 (16–20)
**Performance**	18.5 (14–20)	20 (17–20)	18 (15–20)	18 (16–20)	17 (16–20)	16 (5–19)
**Effort**	1.5 (1–6.25)	1 (1–3.5)	2 (1–7)	8 (3–12)	4 (2–7)	10 (8–10)
**Frustration**	2.5 (1–5.5)	2 (1–3)	2 (1–4)	4 (1–8.5)	2 (1–3)	5 (2–17)

Descriptive statistics for the parameters of the NASA task load index separated by the patient’s initial NACA score. The data are provided as medians and IQR. Abbreviations: IQR, Interquartile range; N, number of cases. Abbreviations and Legend: NACA I, no intervention by an emergency physician necessary; NACA II, slight disturbance, intervention necessary, case can be handled ambulatory; NACA III, moderate to severe, but not life-threatening disturbance, stationary treatment required; NACA IV, development of life-threatening condition cannot be excluded, Intervention by an emergency physician necessary, transport to resuscitation area; NACA V, acute danger for the patient, rapid development to a live-threatening injury possible; NACA VI, and life-threatening injury, respiratory or cardiac arrest with CPR during the sortie, respectively. Abbreviations: IQR, inter-quartile range.

**Table 3:** Descriptive statistics for the physiological workload indicators.

		Patients initial NACA score					
		I	II	III	IV	V	VI
	N	1	13	17	12	3	3
**PeEn**	Mean	10.2	11.0	11.0	11.0	10.8	12.2
	SD	-	0.69	0.91	0.54	0.37	0.47
**Mean HR**	Mean	86.9	85.6	87.5	78.0	88.0	96.8
	SD	-	13.0	12.6	16.8	17.4	13.0

Means and standard deviation for physiological correlates of physician workload separated by the patient’s initial NACA score. The data are provided as means and standard deviation. Abbreviations: Std. dev, Standard deviation; N, number of cases. Abbreviations: PeEn, Permutation Entropy; Mean HR, mean heart rate; SD., standard deviation.

**Table 4:** General linear mixed models for emergency physician subjective and physiological workload indicators.

		NASA Task Load Index							
		Mental	Physical	Temporal	Performance	Effort	Frustration	PeEn	Mean HR
p-value of the linear model		<0.001**	0.14	<0.001**	0.25	<0.001**	0.04*	0.10	0.19
**Reference**		2	1	3	18.5	1.5	2.5	10.2	86.9
Coefficients for the respective NACA scores compared to NACA I	II	-0.001 (p = 1.0)	-0.044 (p = 0.978)	-0.829 (p = 0.676)	1.331 (p = 0.361)	0.002 (p = 0.999)	-0.285 (p = 0.868)	0.9 (p = 0.214)	-4.8 (p = 0.567)
	III	1.617 (p = 0.339)	0.870 (p = 0.579)	-0.153 (p = 0.937)	0.8422 (p = 0.552)	1.227 (p = 0.445)	0.262 (p = 0.876)	1.0 (p = 0.156)	-7.7 (p = 0.361
	IV	4.563 (p = 0.012)*	-0.134 (p = 0.936)	3.825 (p = 0.062)	0.980 (p = 0.511)	3.409 (p = 0.046)*	1.819 (p = 0.303)	1.1 (p = 0.137)	-7.7 (p = 0.362)
	V	2.285 (p = 0.257)	2.221 (p = 0.235)	3.376 (p = 0.131)	1.489 (p = 0.375)	1.672 (p = 0.381)	0.396 (p = 0.841)	0.8 (p = 0.328)	-2.4 (p = 0.802)
	VI	7.789 (p = 0.001)*	3.390 (p = 0.103)	10.376 (p<0.001)**	-1.978 (p = 0.288)	6.702 (p = 0.002)*	4.749 (p = 0.032)*	2.1 (p = 0.011)*	5.6 (p = 0.571)

General linear mixed models for the subjective (NASA-tlx., NASA task load index) and physiological (PeEn, Mean HR) indicators of workload. For NACA I the median NASA-tlx scores (see Table 2) or the respective mean PeEn and mean HR (see Table 3) among all participants for NACA I are provided as a reference. For NACA II to VII the fixed coefficients from the general linear mixed model are provided. Significant p-values are marked with asterisks (* for p<0.05 and ** for p<0.001). Abbreviations: PeEn, Permutation Entropy; Mean HR, mean heart rate.

### Mean code related strain

99 different alarm codes occurred during the 216 sorties. For all of them, subjective strain ratings were obtained from all 20 physicians. The median strain for all 99 alarm codes was 1.85 (interquartile range 1.65–2.45). Table 5 provides the top ten alarm codes that caused the highest and lowest subjective anxiety level, respectively. Mean strain, interquartile range, and frequency of all alarm codes are provided in supporting information file S1 Table.

**Table 5:** Alarm codes that caused highest and lowest mean strain.

Alarm Code	Mean Strain (IQR)	Strain Ranking
Analgesia	1.1 (1–1)	1
Fracture (Finger)	1.1 (1–1)	2
Discus herniation	1.2 (1–1)	3
Hyperglycemia	1.3 (1–1)	4
Fracture (Arm)	1.3 (1–2)	5
Hypertonia	1.3 (1–1.75)	6
Shoulder luxation	1.3 (1–2)	7
Hypoglycemia	1.4 (1–2)	8
Alcohol intoxication	1.4 (1–2)	9
Sinus tachycardia	1.4 (1–2)	10
CPR	2.6 (2–3.75)	72
Ongoing CPR on scene	2.8 (2–4)	89
Unconsciousness	2.9 (2–4)	90
Suicide, *Knife*	2.9 (2–4)	91
Child, fell off tree, analgesia	2.9 (2–4)	92
Car accident, 2 victims	2.9 (2–3.75)	93
After almost drowning	3.0 (2–4)	94
Dyspnea (Child)	3.5 (3–4)	95
Car accident (Child)	3.6 (3–4)	96
Somnolence (Child)	3.7 (3–4)	97
Child in the water	4.0 (4–5)	98
Unconsciousness (Child)	4.2 (4–5)	99

Verbal description of the top 10 alarm codes that caused the highest and lowest mean anxiety within the emergency physicians (N = 20) and the alarm codes including cardiopulmonary resuscitation, respectively. The alarm codes were ranked on a Likert-scale ranging from ‘not nervous at all’ (1) to ‘very nervous’ (5). Their names were ‘not nervous at all’, ‘slightly nervous’, ‘moderately nervous’, ‘rather nervous’, and ‘very nervous’, respectively. IQR, Interquartile Range.

## Discussion

The aim of this study was to investigate the association among patient’s initial NACA scores and subjective as well as physiological indicators of workload during pre-hospital emergency care. As hypothesized, subjective workload in the subscales *mental* and *temporal demands* as well as in *effort* and *frustration* of the NASA-tlx was significantly associated with the patient’s initial NACA score.

CPR (NACA VI) was significantly linked to workload in *temporal demands* (p<0.001), *mental demands* (p = 0.001), *effort* (p = 0.002), and *frustration* (p = 0.03). Although not significant in the ANOVA of the GLMM but in line with what we expected, CPR had the highest coefficients for *physical demands* and *performance* as well as the physiological workload indicators PeEn and mean HR, respectively. The increase in workload during sorties that included CPR seems to be important as lower performance during CPR has been associated with individual stress and the social dynamics of the resuscitation situation.[30–33] Moreover, the peak of workload indicators during CPR in our study is in line with results reported by Havel and co-workers who found perceived exertion (Borg’s Rating of Perceived Exertion, RPE) and systolic blood pressure to increase constantly during CPR.[34] Correspondingly, CPR is frequently declared a very stressful situation for all team members[32, 33] and survival rates after CPR were related to the time of day.[35]

Remarkably, the dimension *performance* was not associated with the patient’s initial NACA score. Consistently, in our research on anesthetists’ workload in the operation theatre NASA-tlx *performance* measures showed a low area under the curve (AUC, 0.508) regarding their ability to separate levels of workload between the phases of induction and maintenance of anesthesia.[9] However, Leedal and Smith stated that performance in the anesthetic environment is difficult to measure because the outcome is influenced by external factors and individuals might compensate disadvantages in performance by increased effort.[2]

The indicators of physiological workload, PeEn (p = 0.10) and mean HR (p = 0.19), did not reach statistical significance according to the ANOVA of the GLMM. However, in pair-wise comparisons, mean PeEn differed between NACA scores (Table 2). This is in line with our previous work, where HRV metrics provided construct validity as correlates of workload in the operation theatre[9] as well as during pre-hospital emergency care.[15] Among them, especially PeEn provided a high AUC (0.993) to separate workload levels before the alarm and during primary patient care.[15] Facing the little number of NACA VI cases, the lack of statistical significance for PeEn in the present analysis might be caused by a type II error.

Mean HR, in contrast, although highest during CPR, did not provide consistent changes in the pair-wise comparisons of NACA scores (Table 3). Accordingly, during pre-hospital emergency care, mean HR showed an AUC of only 0.558 when comparing different workload levels.[15] Likewise, the physician’s mean HR seemed to be independent of the influence of NACA scores in this study. This was surprising since the AUC of mean HR to discriminate the induction of general anesthesia from maintenance in the operation theatre setting was 0.826.[9] As discussed in detail elsewhere, the poor performance of mean HR during pre-hospital emergency care might be due to high variations among the physician’s baseline heart rate.[15] Nevertheless, a variety of studies indicates a correlation between workload and heart rate.[9, 36] Notably, the findings of the present study are in contrast to the results of Schoenenberger and co-workers who found *physical demands* to be the factor that is most influenced by patient characteristics which–in our study–should be reflected by the NACA score.[16] According to Leedal and Smith’s review, however, physiological workload indicators do not necessarily correlate with workload, since their use is based on the assumption that changes in workload causes changes to the body.[2]

NASA-tlx analyses have been used to quantify and analyze workload in a variety of medical settings, e.g. analysis of workload differences among radiation oncology professional subgroups,[4] commanding a joint emergency exercise,[5] analysis of workload caused by noise in the operating theatre.[6] Recently, Parson and colleagues demonstrated the validity of the NASA-tlx scale for workload measurement during pediatric resuscitation.[8] However, to our knowledge, this analysis is the first to evaluate the NASA-tlx in pre-hospital emergency care.

The analysis of alarm codes unveiled that sorties which included ‘child’ as part of the briefing-alarm code caused the highest strain within the participants (Table 5). Surprisingly, CPR and ongoing CPR on the scene were not among the ten alarm codes provoking the highest strain. This might reflect that CPR *per se* is a well-trained scenario that does not cause considerable strain within the physician *a priori*, but the *particular* CPR in an unknown environment during pre-hospital emergency care does cause high amounts of workload. Moreover, there seems to be further need for training of pediatric emergencies embedded in the emergency physician’s curriculum since they cause high levels of strain. [16–18] Nevertheless, it has to be pointed out that CPR is an independent category in the NACA scoring system, whereas the medical condition of children during emergency care may range from NACA I to NACA VII. Hence, further research on the correlation of briefing-alarm codes and NACA scores as well as physicians’ workload is needed.

NACA scores have been commonly used to characterize patients;[23, 24, 37–40] with good inter-rater reliability.[22] Still, the NACA scoring-system has been criticized for its lack of objectivity,[39] its dependency on the time of classification[37] and its association with the emergency physician’s experience.[40]

This study has some limitations. First, due to the low absolute number of CPRs, the number of cases with a NACA score of 6 was low (N = 7). This increases the risk for both type I and type II errors. Additionally, physiological correlates of workload were available for a smaller number (N = 13) of emergency physicians, only. As this study was exploratory, an a priori calculation of power size was not possible. Second, as already mentioned above, the patient’s initial NACA score itself is influenced by experience, time and other subjective parameters and might therefore also function as a surrogate of subjective workload. Third, another important consideration is the heterogeneity of the study group, which in our opinion is both strength and limitation of the study. Not all participants worked at the department of anesthesiology and the IQR for experience in emergency medicine ranged from 0.9 to 15.7 years. Nevertheless, as we intended to investigate the workload measures in pre-hospital care, the heterogeneity might also improve reproducibility as 25% of the emergency physicians in Germany are residents and an overall of only 50% of the pre-hospital care providers works in anesthesia. Furthermore, Byrne and co-workers found larger differences between subjects of the same rather than different experience[29] and DeAnda and colleagues were not able to find differences between second year residents and experienced anesthesiologists in their response to simulated critical incidents.[28] Last, the anxiety regarding sorties that include the treatment of children might be biased by the fact that the participating physicians work at a university hospital, where the pediatric department is not located on the main campus and therefore critically ill children are not part of their daily clinical work.

## Conclusion

We conclude that this study provides further evidence for the validity of the NASA-tlx in the assessment of emergency physicians’ subjective workload during pre-hospital emergency care. Specifically, the dimensions *mental demand*, *temporal demand*, *effort* and *frustration* were associated with the patient’s NACA score. As indicated by all parameters including PeEn and HR, workload was highest during CPR. The analysis of briefing-code related strain showed that the participants’ strain was especially high when children were involved.

## Supporting information

S1 Table HRV, NACA scores and alarm codes. This table contains the data used for statistical analysis.
(XLSX)

S1 Text NASA task load query. This file contains the German query used for acquisition of the NASA task load index as well as an English translation.
(DOCX)
