# Supplementary material for: The impact of the patient’s initial NACA score on subjective and physiological indicators of workload during pre-hospital emergency care
Source: PLoS One. 2018 Aug 9;13(8):e0202215. doi: 10.1371/journal.pone.0202215 (PMC6084954; doi:10.1371/journal.pone.0202215)
Supplement: S1 Text — This file contains the German query used for acquisition of the NASA task load index as well as an English translation. (DOCX) [file pone.0202215.s002.docx]

**S1 Text. NASA task load query.** This file contains the German query used for acquisition of the NASA task load index as well as an English translation.

## Query regarding YOUR emergency sortie

| D | D | M | M | 2015 |
| --- | --- | --- | --- | --- |

Date of the sortie:

Alarm time:

| H | H | S | S |
| --- | --- | --- | --- |

Alarm code (according to briefing-alarm code):

___________________________________

Please judge these statements according to your workload during the sortie (please cross):

**How mentally demanding was the task?**

|  |  |  |  |  |  |  |  |  |  |  |  |  |  |  |  |  |  |  |  |
| --- | --- | --- | --- | --- | --- | --- | --- | --- | --- | --- | --- | --- | --- | --- | --- | --- | --- | --- | --- |

Very Low Very High

**How physically demanding was the task?**

|  |  |  |  |  |  |  |  |  |  |  |  |  |  |  |  |  |  |  |  |
| --- | --- | --- | --- | --- | --- | --- | --- | --- | --- | --- | --- | --- | --- | --- | --- | --- | --- | --- | --- |

Very Low Very High

**How hurried or rushed was the pace of the task?**

|  |  |  |  |  |  |  |  |  |  |  |  |  |  |  |  |  |  |  |  |
| --- | --- | --- | --- | --- | --- | --- | --- | --- | --- | --- | --- | --- | --- | --- | --- | --- | --- | --- | --- |

Very Low Very High

**How successful were you in accomplishing what you were asked to?**

|  |  |  |  |  |  |  |  |  |  |  |  |  |  |  |  |  |  |  |  |
| --- | --- | --- | --- | --- | --- | --- | --- | --- | --- | --- | --- | --- | --- | --- | --- | --- | --- | --- | --- |

Very Low Very High

**How hard did you have to work to accomplish your performance level?**

|  |  |  |  |  |  |  |  |  |  |  |  |  |  |  |  |  |  |  |  |
| --- | --- | --- | --- | --- | --- | --- | --- | --- | --- | --- | --- | --- | --- | --- | --- | --- | --- | --- | --- |

Very Low Very High

**How stressed or irritated did you feel?**

|  |  |  |  |  |  |  |  |  |  |  |  |  |  |  |  |  |  |  |  |
| --- | --- | --- | --- | --- | --- | --- | --- | --- | --- | --- | --- | --- | --- | --- | --- | --- | --- | --- | --- |

Very Low Very High

## FRAGEBOGEN ZUM EINSATZ

| D | D | M | M | 2015 |
| --- | --- | --- | --- | --- |

Datum des Einsatzes:

Uhrzeit des Einsatzbeginns:

| H | H | S | S |
| --- | --- | --- | --- |

Meldebild (laut Fax): ___________________________________

Bitte beurteilen Sie folgende Aussagen in Bezug auf Ihre Belastung während des Einsatzes (bitte Ankreuzen):

**Wie hoch war die mentale Anstrengung bei der Tätigkeit?**

|  |  |  |  |  |  |  |  |  |  |  |  |  |  |  |  |  |  |  |  |
| --- | --- | --- | --- | --- | --- | --- | --- | --- | --- | --- | --- | --- | --- | --- | --- | --- | --- | --- | --- |

sehr niedrig sehr hoch

**Wie hoch war die körperliche Anstrengung bei der Tätigkeit?**

|  |  |  |  |  |  |  |  |  |  |  |  |  |  |  |  |  |  |  |  |
| --- | --- | --- | --- | --- | --- | --- | --- | --- | --- | --- | --- | --- | --- | --- | --- | --- | --- | --- | --- |

sehr niedrig sehr hoch

**Wie groß war der Zeitdruck bei der Tätigkeit?**

|  |  |  |  |  |  |  |  |  |  |  |  |  |  |  |  |  |  |  |  |
| --- | --- | --- | --- | --- | --- | --- | --- | --- | --- | --- | --- | --- | --- | --- | --- | --- | --- | --- | --- |

sehr niedrig sehr hoch

**Wie erfolgreich haben Sie die Aufgabe gelöst?**

|  |  |  |  |  |  |  |  |  |  |  |  |  |  |  |  |  |  |  |  |
| --- | --- | --- | --- | --- | --- | --- | --- | --- | --- | --- | --- | --- | --- | --- | --- | --- | --- | --- | --- |

sehr niedrig sehr hoch

**Wie sehr haben Sie sich angestrengt dafür?**

|  |  |  |  |  |  |  |  |  |  |  |  |  |  |  |  |  |  |  |  |
| --- | --- | --- | --- | --- | --- | --- | --- | --- | --- | --- | --- | --- | --- | --- | --- | --- | --- | --- | --- |

sehr niedrig sehr hoch

**Wie unsicher, entmutigt, irritiert, gestresst oder enttäuscht waren Sie?**

|  |  |  |  |  |  |  |  |  |  |  |  |  |  |  |  |  |  |  |  |
| --- | --- | --- | --- | --- | --- | --- | --- | --- | --- | --- | --- | --- | --- | --- | --- | --- | --- | --- | --- |

sehr niedrig
